# Predictors of non-persistence in women with overactive bladder syndrome

**DOI:** 10.1038/s41598-024-58036-4

**Published:** 2024-03-29

**Authors:** Sheng-Mou Hsiao

**Affiliations:** 1 Department of Obstetrics and Gynecology, Far Eastern Memorial Hospital, No. 21, Sec. 2, Nanya S. Rd., Banqiao Dist., New Taipei City, Taiwan; 2 Graduate School of Biotechnology and Bioengineering, Yuan Ze University, Taoyuan, Taiwan; 3 Department of Obstetrics and Gynecology, National Taiwan University Hospital, Taipei, Taiwan

**Keywords:** Medication adherence, Mirabegron, Solifenacin succinate, Urinary bladder, Overactive, Urodynamics, Medical research, Risk factors, Signs and symptoms, Urology

## Abstract

Persistence is important for the success in the treatment of women with overactive bladder syndrome (OAB). We aimed to identify the predictors of non-persistence in women with OAB after first-line medical treatment. All consecutive women with OAB (n = 608), who underwent urodynamic studies and received first-line medical treatment (5 mg of solifenacin or 25 mg of mirabegron per day) in a referral medical center, were reviewed. Mirabegron (hazard ratio [HR] = 0.711) was associated with a higher persistence rate, compared to solifenacin. Mirabegron treatment (HR = 0.269) was less likely to switch medication; however, a high Urogenital Distress Inventory score (HR = 1.082) was more likely to switch medication. Furthermore, old age (HR = 1.050, especially for ≥ 75 years) and high voided volume (dL, HR = 1.420, especially for voided volume ≥ 250 ml) were associated with added medication at follow-up. Additionally, women with low parity (HR = 0.653, especially for parity ≤ 3) and a low Incontinence Impact Questionnaire (IIQ-7) score (HR = 0.828, especially for IIQ-7 score ≤ 7) were associated with improvement without medication. In conclusion, mirabegron can be considered as the first frontline treatment to increase the persistence rate and decrease the rate of switched medications, compared to solifenacin. In addition, combination therapy or higher-dose monotherapy could be used as the first front-line treatment for women ≥ 75 years of age or with ≥ 250 ml of voided volume.

## Introduction

Overactive bladder syndrome (OAB) is characterized by urinary urgency, frequency, and nocturia. Both antimuscarinics and beta-3 agonists are considered first-line medical treatments for OAB. Several antimuscarinics (for example, solifenacin and tolterodine) are currently marketed for OAB treatment. Solifenacin has a moderate selectivity for the M3 receptor over the M2 receptor. Beta-3 agonists relax the detrusor muscle during the bladder storage phase and increase bladder capacity. Mirabegron is the first beta-3 agonist approved for OAB treatment. Mirabegron has been reported to have similar efficacy to antimuscarinics^1^; however, the beta-3 agonist is associated with less bothersome adverse effects, such as dry mouth^2^. However, the question of whether solifenacin and mirabegron have different persistence rates remains undetermined^3–6^.

Persistence is generally referred to the overall duration of drug therapy. OAB typically requires long-term persistence with medical therapy^7^. Patients who persist in taking OAB medication have a significant improvement in OAB symptoms compared to non-persistent^8^. Discontinuation of medication or lost follow-up had been used as the definition of non-persistence^9^; switched medications and added medication were excluded as non-persistence^10^.

Lack of efficacy, improvement in symptoms, adverse effects, insurance limits, cost concerns, and inconvenience are considered causes of non-persistence^5,10,11^. Knowledge of non-persistence is important to improve persistence and personal precision treatment.

In addition, urodynamic studies might be helpful in the treatment of complex storage diseases such as OAB^12^. A Female Urgency, Trial of Urodynamics as Routine Evaluation (FUTURE) study was conducted to evaluate whether routine urodynamics improves treatment results in women with refractory OAB^13^.

Due to the positive association between the persistence of OAB medication and therapeutic efficacy^8^, the issue of improving persistence is important. Therefore, this study aimed to analyze clinical and urodyamic predictors of non-persistence.

## Results

Between July 2010 and December 2020, a total of 608 women were reviewed in this study. Except age, OAB-wet and pad weight, there were no differences between solifenacin and mirabegron (Table 1).

**Table 1:** Baseline data of women with overactive bladder syndrome (n = 608).

Variables	Mirabegron (n = 212)	Solifenacin (n = 396)	*P*^a^
Age (years)	58.5 ± 14.0	53.4 ± 11.9	< 0.001
Parity	2.4 ± 1.3	2.4 ± 1.3	0.306
BMI (kg/m^2^)	19.8 ± 1.7	19.7 ± 1.6	0.489
SUI	72 (34)	154 (39)	0.231
OAB-wet	127 (60)	204 (52)	0.048
UDI-6	6.7 ± 3.6	6.2 ± 3.2	0.162
IIQ-7	7.2 ± 5.5	6.7 ± 5.1	0.481
Pad weight (g)	36.8 ± 65.2	17.9 ± 48.5	< 0.001
Qmax (mL/s)	23.9 ± 13.0	22.3 ± 12.3	0.220
VV (dL)	2.76 ± 1.44	2.76 ± 1.38	0.769
PVR (dL)	0.97 ± 0.72	1.07 ± 0.86	0.299
SD (dL)	2.69 ± 1.20	2.66 ± 1.03	0.548
PdetQmax (cmH_2_O)	43 ± 36	38 ± 24	0.385
MUCP (cmH_2_O)	75 ± 37	80 ± 36	0.072
FPL (mm)	30 ± 11	31 ± 11	0.436
PTR (%)	82 ± 43	87 ± 40	0.352
Non-persistence	66 (31)	161 (41)	0.008^b^
Switched medications	17 (8)	72 (18)	< 0.001^b^
Added medications	12 (6)	6 (2)	0.026^b^
Improvement without medication	8 (4)	11 (3)	0.809^b^

Values were presented with mean ± standard deviation or number (percentage).

*BMI* body mass index; *FPL* functional profile length; *IIQ-7* Incontinence Impact Questionnaire; *MUCP* maximum urethral closure pressure; *OAB* overactive bladder syndrome; *PdetQmax* detrusor pressure at maximum flow rate; *PTR* pressure transmission ratio at maximum urethral pressure; *PVR* postvoid residual volume; *Qmax* maximum flow rate; *SD* the volume at strong desire to void; *SUI* stress urinary incontinence; *UDI-6* short form of Urinary Distress Inventory; *VV* voided volume.

^a^Wilcoxon rank sum test, chi-square test or Fisher’s exact test.

^b^Log-rank test.

**Figure 1:**
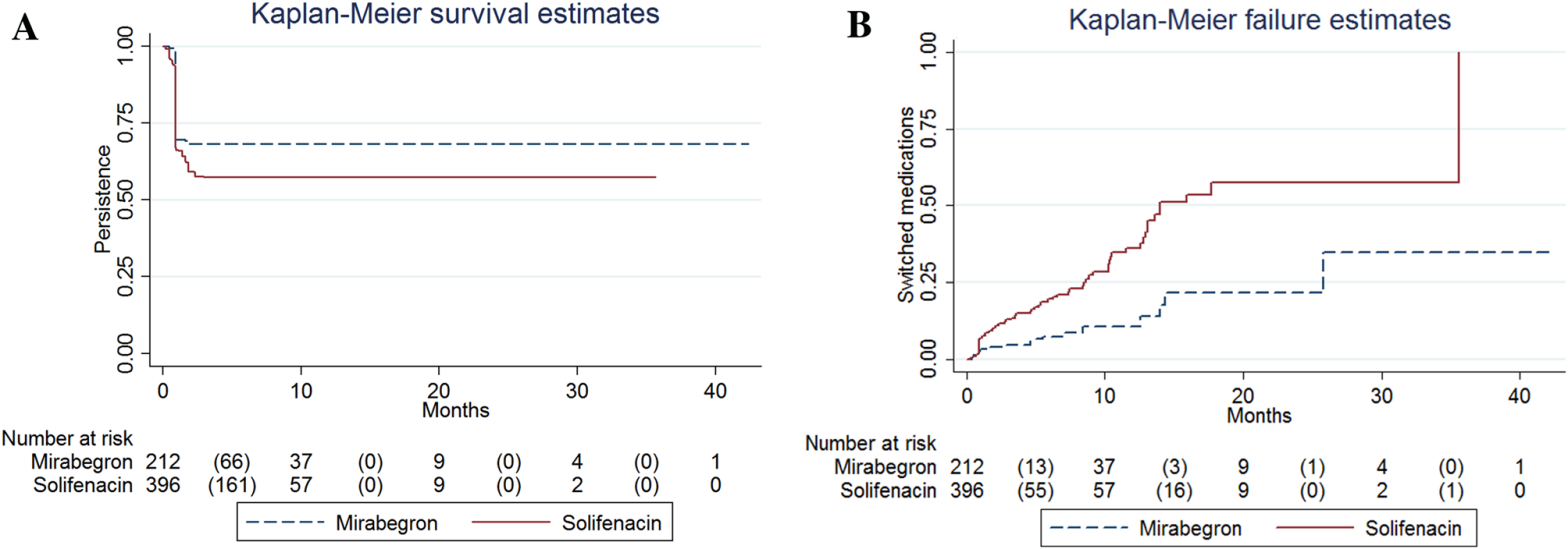
Comparison of (**A**) persistence and (**B**) switched medications probabilities between mirabegron and solifenacin.

There was a statistical difference in the persistent curves between the mirabegron and solifenacin groups (log-rank test, *p* = 0.008, Fig. 1A). The multivariable Cox regression model also showed that mirabegron (hazard ratio [HR] = 0.711, *p* = 0.019) was the only predictor of non-persistence (Table 2). OAB-wet was not a predictor of non-persistence (HR = 1.047, *p* = 0.729, Table 2).

**Table 2:** Univariate and multivariable Cox proportional hazards model to predict non-persistence in women with overactive bladder syndrome (n = 608).

Variables	Univariate			Multivariable		
	HR	95% CI	*P*^a^	HR	95% CI	*P*^b^
Mirabegron	0.711	0.534–0.946	0.019	0.711	0.534–0.946	0.019
Age (years)	0.992	0.983–1.003	0.142	–	–	–
Parity	0.974	0.881–1.077	0.610	–	–	–
BMI (kg/m^2^)	0.984	0.901–1.075	0.723	–	–	–
SUI	1.259	0.967–1.639	0.087	–	–	–
OAB-wet	1.047	0.806–1.360	0.729	–	–	–
UDI-6	1.012	0.963–1.063	0.644	–	–	–
IIQ-7	0.999	0.968–1.031	0.959	–	–	–
Pad weight (g)	1.000	0.998–1.002	1.000	–	–	–
Qmax (mL/s)	1.002	0.992–1.012	0.721	–	–	–
VV (dL)	1.000	0.913–1.096	0.998	–	–	–
PVR (dL)	0.974	0.823–1.152	0.756	–	–	–
SD (dL)	0.972	0.859–1.100	0.655	–	–	–
PdetQmax (cmH_2_O)	1.000	0.995–1.006	0.945	–	–	–
MUCP (cmH_2_O)	1.001	0.997–1.004	0.742	–	–	–
FPL (mm)	1.000	0.989–1.012	0.971	–	–	–
PTR (%)	1.000	0.997–1.004	0.852	–	–	–

*HR* hazard ratio; *CI* confidence interval. The other abbreviations are the same as in Table 1.

^a^Univariate Cox proportional hazards model.

^b^Multivariable backward stepwise Cox proportional hazards model was performed using all variables in the univariate analysis with *p* < 0.10 until all remaining variables with *p* < 0.10.

Mirabegron (HR = 0.269, *p* < 0.001) was associated with a lower incidence of switched medications (Fig. 1B, Table 3). Furthermore, a higher baseline Urogenital Distress Inventory (UDI-6) score^14^ (HR = 1.082,* p* = 0.029) was associated with a higher incidence of switched medications (Table 3). The UDI-6 score ≥ 10 was the optimal cutoff value for predicting switched medications, with an area under the receiver operating characteristic curve (AUC) of 0.534 (95% confidence interval [CI] = 0.452 to 0.616; sensitivity = 26.6%, specificity = 83.6%).

**Table 3:** Univariate and multivariable Cox proportional hazards model to predict switched medications in women with overactive bladder syndrome (n = 608).

Variables	Univariate			Multivariable		
	HR	95% CI	*P*^a^	HR	95% CI	*P*^b^
Mirabegron	0.334	0.196–0.569	< 0.001	0.269	0.152–0.477	< 0.001
Age (years)	0.997	0.982–1.013	0.744	–	–	–
Parity	1.041	0.896–1.210	0.597	–	–	–
BMI (kg/m^2^)	0.991	0.864–1.135	0.891	–	–	–
SUI	1.468	0.959–2.247	0.077	–	–	–
OAB-wet	1.311	0.850–2.021	0.221	–	–	–
UDI-6	1.061	0.989–1.138	0.097	1.082	1.008–1.162	0.029
IIQ-7	1.021	0.975–1.068	0.378	–	–	–
Pad weight (g)	0.997	0.993–1.002	0.252	–	–	–
Qmax (mL/s)	0.996	0.978–1.014	0.679	–	–	–
VV (dL)	0.976	0.843–1.129	0.743	–	–	–
PVR (dL)	1.031	0.767–1.386	0.838	–	–	–
SD (dL)	0.814	0.660–1.004	0.055	–	–	–
PdetQmax (cmH_2_O)	1.002	0.996–1.010	0.430	–	–	–
MUCP (cmH_2_O)	0.997	0.991–1.003	0.343	–	–	–
FPL (cm)	1.004	0.985–1.023	0.700	–	–	–
PTR (%)	0.999	0.994–1.005	0.782	–	–	–

The abbreviations are the same as in Tables 1 and 2.

^a^Univariate Cox proportional hazards model.

^b^Multivariable backward stepwise Cox proportional hazards model was performed using all variables in the univariate analysis with *p* < 0.10 until all remaining variables with *p* < 0.10.

Old age (HR = 1.050, *p* = 0.009) and larger voided volume (dL, HR = 1.420,* p* = 0.001) were associated with a higher incidence of added medications (Table 4). Age ≥ 75 years was the optimal cut-off value for predicting added medications, with an AUC of 0.727 (95% CI = 0.611 to 0.842; sensitivity = 33.3%, specificity = 92.2%). Voided volume ≥ 2.50 dL (i.e., 250 mL) was the optimal cut-off value for predicting added medications, with an AUC of 0.657 (95% CI = 0.541 to 0.772; sensitivity = 76.5%, specificity = 50.0%).

**Table 4:** Univariate and multivariable Cox proportional hazards model to predict added medications in women with overactive bladder syndrome (n = 608).

Variables	Univariate			Multivariable		
	HR	95% CI	*P*^a^	HR	95% CI	*P*^b^
Mirabegron	2.894	1.083–7.732	0.034	2.592	0.861–7.808	0.090
Age (years)	1.053	1.018–1.090	0.003	1.050	1.012–1.089	0.009
Parity	1.277	0.949–1.718	0.107	–	–	–
BMI (kg/m^2^)	1.136	0.864–1.493	0.362	–	–	–
SUI	0.639	0.210–1.948	0.431	–	–	–
OAB-wet	2.032	0.723–5.712	0.179	–	–	–
UDI-6	1.038	0.898–1.199	0.615	–	–	–
IIQ-7	0.913	0.818–1.019	0.104	–	–	–
Pad weight (g)	1.007	1.002–1.012	0.009	–	–	–
Qmax (mL/s)	1.002	0.964–1.042	0.915	–	–	–
VV (dL)	1.396	1.107–1.759	0.005	1.420	1.149–1.755	0.001
PVR (dL)	0.987	0.501–1.941	0.969	–	–	–
SD (dL)	1.350	0.963–1.892	0.082	–	–	–
PdetQmax (cmH_2_O)	0.984	0.960–1.010	0.219	–	–	–
MUCP (cmH_2_O)	0.999	0.986–1.011	0.843	–	–	–
FPL (cm)	0.973	0.922–1.026	0.311	–	–	–
PTR (%)	1.004	0.993–1.015	0.508	–	–	–

The abbreviations are the same as in Tables 1 and 2.

^a^Univariate Cox proportional hazards model.

^b^Multivariable backward stepwise Cox proportional hazards model was performed using all variables in the univariate analysis with *p* < 0.10 until all remaining variables with *p* < 0.10.

Mirabegron was not associated with improvement without treatment (Table 5). However, low parity (HR = 0.653, *p* = 0.020) and low baseline Incontinence Impact Questionnaire (IIQ-7) score^14^ (HR = 0.828, *p* = 0.007) were associated with improvement without medication (Table 5). Parity ≤ 3 was the optimal cutoff value for predicting improvement without medication, with an AUC of 0.692 (95% CI = 0.587 to 0.797; sensitivity = 43.4%, specificity = 89.5%). The baseline IIQ-7 score ≤ 7 was the optimal cut-off value for predicting improvement without medication, with an AUC of 0.705 (95% CI = 0.602 to 0.808; sensitivity = 47.1%, specificity = 83.3%).

**Table 5:** Univariate and multivariable Cox proportional hazards model to predict improvement without medication in women with overactive bladder syndrome (n = 608).

Variables	Univariate			Multivariable		
	HR	95% CI	*P*^a^	HR	95% CI	*P*^b^
Mirabegron	1.119	0.449–2.788	0.810	–	–	–
Age (years)	0.967	0.934–1.002	0.065	–	–	–
Parity	0.591	0.403–0.866	0.007	0.653	0.443–0.933	0.020
BMI (kg/m^2^)	0.959	0.719–1.279	0.774	–	–	–
SUI	0.890	0.338–2.345	0.813	–	–	–
OAB-wet	0.771	0.313–1.898	0.571	–	–	–
UDI-6	0.885	0.746–1.050	0.162	–	–	–
IIQ-7	0.829	0.723–0.950	0.007	0.828	0.723–0.949	0.007
Pad weight (g)	0.978	0.947–1.010	0.181	–	–	–
Qmax (mL/s)	1.010	0.976–1.046	0.551	–	–	–
VV(dL)	0.918	0.652–1.292	0.622	–	–	–
PVR (dL)	1.062	0.596–1.891	0.838	–	–	–
SD (dL)	1.304	0.919–1.849	0.137	–	–	–
PdetQmax (cmH_2_O)	1.002	0.988–1.016	0.763	–	–	–
MUCP (cmH_2_O)	1.005	0.994–1.016	0.390	–	–	–
FPL (cm)	1.003	0.965–1.043	0.861	–	–	–
PTR (%)	1.004	0.993–1.015	0.457	–	–	–

The abbreviations are the same as in Tables 1 and 2.

^a^Univariate Cox proportional hazards model.

^b^Multivariable backward stepwise Cox proportional hazards model was performed using all variables in the univariate analysis with *p* < 0.10 until all remaining variables with *p* < 0.10.

The details of the switched medications and the added medications are shown in Table 6.

**Table 6:** Switched/added medications in the mirabegron and solifenacin groups.

First frontline medication	Switched medications at follow-up (n = 89)	n	First frontline medication	Added medications at follow-up (n = 18)	n
Mirabegron	Solifenacin	12	Mirabegron	Oxybutynin ER	8
	Imipramine	3		Imipramine	4
	Oxybutynin ER	1			
	Bethanechol	1			
Solifenacin	Mirabegron + oxybutynin ER	7	Solifenacin	Oxybutynin ER	3
	Tolterodine + imipramine	2		Imipramine	2
	Mirabegron	36		Mirabegron	1
	Tolterodine	12			
	Oxybutynin ER	7			
	Bethanechol	4			
	Desmopressin	3			
	Imipramine	1			

## Discussion

In this study, women who received mirabegron treatment tended to have a higher incidence of persistence (Fig. 1A, Table 2). Similarly, a study from the national cohort database of Korea found that mirabegron has a longer persistence than antimuscarinics^4^. The PERSPECTIVE (a Prospective Non-interventional Registry Study of Patients Initiating a Course of Drug Therapy for Overactive Bladder) study revealed that the persistence was longer for mirabegron compared with antimuscarinics ^5^. Chapple et al. also reported that mirabegron had a higher persistence rate compared to antimuscarinics^6^. Nazir et al. and Yeowell et al. had similar findings on the superiority of mirabegron in persistence^15,16^. However, Lee et al. reported that there is no difference in persistence between groups^4^. Sussman et al. reported that persistence rates were similar between solifenacin and mirabegron^17^.

In our study, mirabegron was associated with a lower incidence of switched medications (Fig. 1B, Table 3). The causes of switched medications could include cost consideration, lack of efficacy,or intolerable adverse effects. In Taiwan, the fee for solifenacin and mirabegron was covered by the National Health Insurance. In addition, the same efficacy between mirabegron and solifenacin has been reported^1^. Furthermore, higher adverse effects were observed for antimuscarinic drugs^2^. Thus, the lower adverse effects of mirabegron should contribute to its lower rate of switching medications.

The UDI-6 score (HR = 1.082) is a predictor of switched medications (Table 3). UDI-6 question 2 is the question for urgency incontinence severity. In this study, the UDI-6 score was strongly associated with UDI-6 question 2 (Spearman’s rho = 0.62, *p* < 0.001); and this meant that higher severity of urgency incontinence was associated with higher rate of switched medications. Thus, a lower side effect of the medication (for example, mirabegron) or a higher dose (for example, 50 mg mirabegron) could be used as an initial first-line treatment for women with severe urgency incontinence to decrease the rate of switched medications.

Age (HR = 1.050, especially for those ≥ 75 years) was a predictor of the added medications (Table 4).Similarly, Soda et al. found that old age was associated with continuous treatment, which included added medication^9^. In this study, old age was associated with a higher score on UDI-6 Question 2 (that is, the urgency incontinence, Spearman’s rho = 0.27, *p* < 0.001), and this represents that women of old age had a greater severity of urgency incontinence. Therefore, the medication (5 mg solifenacin or 25 mg mirabegron) might be inadequate in some old women. Higher dose (for example, 50 mg mirabegron) or combination therapy could be used as a first line treatment for old age women.

In our study, high voided volume (HR = 1.420, especially for voided volume ≥ 2.50 dL) was a predictor of added medication (Table 4). High voided volume represents mild severity of OAB^18,19^. Mild severity of OAB was reported to be associated with a poor response to solifenacin^20^. That is, high voided volume might be associated with poor therapeutic response; and women with high voided volume might need higher dose medication or combination therapy to improve treatment response.

In this study, low parity (HR = 0.653, especially for parity ≤ 3) was a predictor of improvement without medication (Table 5). Low parity was associated with mild severity of OAB^21^. Similarly, high parity (HR = 1.81) has been reported to be a predictor of the retreat of OAB symptoms^22^.

In this study, a low IIQ-7 score (HR = 0.828, especially for IIQ-7 score ≤ 7) was a predictor of improvement without medication (Table 5). A low IIQ-7 score means a mild urgency incontinence severity (i.e., IIQ-7 score versus UDI-6 question 2 score (i.e., urgency incontinence score), Spearman's rho = 0.31, *p* = 0.001). Thus, our data represent that women with mild OAB severity have a higher incidence of improvement in symptoms without treatment.

Limitations of this study include retrospective nature and non-randomized. Additionally, the sample size was not equal in both groups. Furthermore, this study covered a period of almost 10 years, and 25 mg of mirabegron was only available since 2016 in our hospital, leading to a significant difference in the median follow-up intervals between solifenacin and mirabegron; and the above might also bias our results. In addition, 50 mg mirabegron was not available in our hospital and our data could not be extrapolated to 50 mg mirabegron. In this study, women with improvement without medication were identified from the medical record. However, the true causes responsible for lost to follow-up include improvement of symptoms or no response to treatment. Therefore, in this study, the percentage of improvement without medication in Table 1 should be underestimated.

In conclusion, mirabegron can be considered as the first frontline treatment to increase the persistence rate and decrease the rate of switched medications, compared to solifenacin. In addition, combination therapy or higher-dose monotherapy could be used as the first front-line treatment for women ≥ 75 years of age or with ≥ 250 ml of voided volume.

## Methods

Between July 2010 and December 2020, medical records of all consecutive OAB women who underwent pretreatment urodynamic studies and then received first-line medication (5 mg of solifenacin or 25 mg of mirabegron per day) were reviewed. Women with coexistent stress urinary incontinence were also included. However, women who underwent a midurethral sling procedure or vaginal laser therapy for coexisting stress urinary incontinence were excluded. The hospital’s Research Ethics Review Committee approved this study (Far Eastern Memorial Hospital, No.110053E, approval date: April 27, 2021). All methods were performed in accordance with relevant guidelines and regulations. The Research Ethics Review Committee agreed that informed consent was not required due to the retrospective nature of this study.

Urodynamic studies were performed on women in a seated position using a Life-Tech six-channel monitor with computer analysis and the Urolab/Urovision System V (Houston, Texas, USA). Urodynamic studies included uroflowmetry, filling cystometry with 35° C distilled water at a rate of 60 ml/s, a pressure flow study, and a stress urethral pressure profile with a strong desire volume of distilled water in the bladder. In addition, a 1 h pad test was performed^23^.

All terminology used in this document is consistent with the standards recommended by the joint report of the International Urogynecological Association and the International Continence Society^24^. All procedures were performed by an experienced technician and the data was interpreted by a single observer to avoid interobserver variability. OAB was defined as the presence of urinary urgency, with or without urgency incontinence, which is generally accompanied by urinary frequency and nocturia^24^. OAB-wet was diagnosed in women who complained of at least one episode of urgency incontinence in the previous month; otherwise, OAB-dry was diagnosed^19^.

Non-persistence after first-line medication was defined as the presence of lost follow-up. The switch of medication was defined as the discontinuation of the first-line OAB medication and the switch to other medications. Improvement without medication was referred to spontaneous improvement of OAB symptoms after first-line medical treatment, and the patients did not need additional OAB medication. The persistence interval was calculated as the time interval from the date of the start of the prescribed medication to the date of loss of follow-up, improvement without medication, or the last follow-up for continuous prescription.

Stata version 11.0 (Stata Corp, College Station, TX) was used for statistical analyzes. Survival curves were generated using the Kaplan–Meier method and differences in survival curves were calculated with the log-rank test. A *p*-value less than 0.05 was considered statistically significant. The multivariable backward stepwise Cox proportional hazards model was used to identify independent predictors using all variables in the univariate analysis with *p* < 0.10 until all remaining variable with *p* < 0.10. A receiver operating characteristic (ROC) curve analysis was performed to identify the optimal cut-off value. The optimal cut-off value was determined by the point on the ROC curve that was closest to the upper left corner.

### Ethical approval

This study was approved by the Research Ethics Committee (Far Eastern Memorial Hospital Research Ethics Review Committee, No.110053E, approval date: 27 April 2021) at Far Eastern Memorial Hospital.

### Informed consent

The Research Ethics Review Committee of Far Eastern Memorial Hospital approved the informed consent waiver.

## Data Availability

The datasets generated and / or analysed during the current study are available from the corresponding author on reasonable request.
